# Organizing research data

**DOI:** 10.1186/1751-0147-53-S1-S2

**Published:** 2011-06-20

**Authors:** Peter Sestoft

**Affiliations:** 1 IT University of Copenhagen, Rued Langgaards Vej 7, DK-2300 Copenhagen S, Denmark

## Abstract

Research relies on ever larger amounts of data from experiments, automated production equipment, questionnaries, times series such as weather records, and so on. A major task in science is to combine, process and analyse such data to obtain evidence of patterns and correlations.

Most research data are on digital form, which in principle ensures easy processing and analysis, easy long-term preservation, and easy reuse in future research, perhaps in entirely unanticipated ways. However, in practice, obstacles such as incompatible or undocumented data formats, poor data quality and lack of familiarity with current technology prevent researchers from making full use of available data.

This paper argues that relational databases are excellent tools for veterinary research and animal production; provides a small example to introduce basic database concepts; and points out some concerns that must be addressed when organizing data for research purposes.

## Database concepts

A database is an organized collection of data. This section presents the most common tool for storing and processing data in modern society: the *relational database*.

### Motivating example

**Figure 1:**
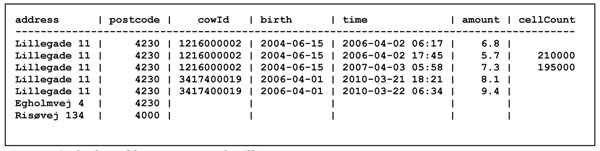
Flat list of farms, cows and milking events

Assume we want to keep records of multiple farms (with address), each with multiple cows (with cow identifier and birth date), and for each cow multiple milking events (with date, time, amount of milk, and possibly somatic cell count). From such data, one can compute many different quantities, such as *total milk production* or *total milk production in each postcode* or *average number of cows per farm* and much more. A simple spreadsheet style solution would use a single table containing all these data, as shown in Figure 1.

However, this is a poor solution for several reasons:

• The address of a farm is repeated for every cow, and the birth date of a cow is repeated for every milking event belonging to that cow. Such redundancy typically leads to inconsistency (e.g. two different addresses recorded for the same farm) and to update problems (e.g. if the street name of a farm is changed).

• If one needs to register a farm before it has a cow, or register a cow before it has a milking event, one must leave some fields blank, which is likely to confuse later processing and analysis.

A better solution is to use a relational database [1]; since 1985 this is the dominant technology for organizing and handling large data sets in production, commerce, finance, research and so on.

### Tables in relational databases

In a relational database the example from Figure 1 would be broken into three separate tables called Farm, Cow and Milk, as shown below. The tables would all be stored in the same *database* inside a *database system*. The database system may simply be Microsoft Access, which is part of the Microsoft Office suite, or it may be the SAS statistical analysis system, and hence the database may reside on the researcher's normal computer. However, if the database is to be shared with others, it is more sensible to keep it on a separate server.

**Figure 2:**
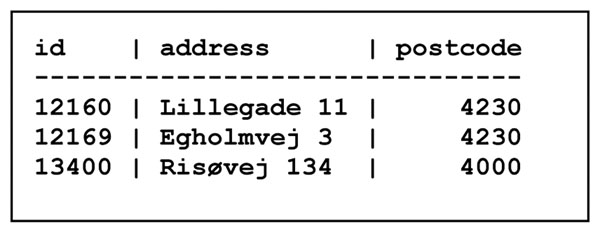
The Farm table

In the Farm table in Figure 2, each line describes a single farm by its unique farm id, address and postcode. The heading lists the *attributes* or *columns* (id, address and postcode) of the table. Each line below it is called a *record* or *row* of the table. The unique farm id is a *key*; a given key must appear at most once in the table. Those database keys are the reason everything (people, cows, supermarket goods) has a number in modern society.

**Figure 3:**
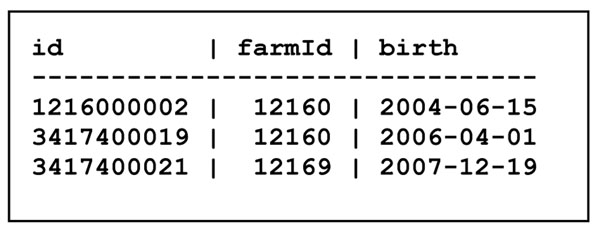
The Cow table

In the Cow table in Figure 3, each record describes a cow: the cow id is the key in the table, the farmId says which farm the cow belongs to, and the birth attribute is the cow's birthdate. A cow's farmId attribute is intended to refer to some farm's id, which is the key in the Farm table; hence the farmId in the Cow table is called a *foreign key*.

**Figure 4:**
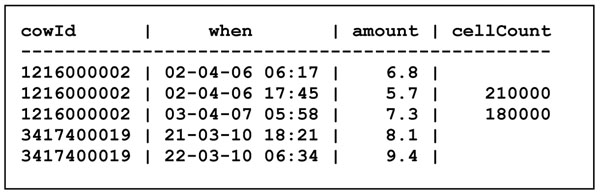
The Milk table

In the Milk table in Figure 4, each record describes a milking event: the cow id together with the date-and-time (the "when" attribute) together constitute the key of the table, the amount of milk obtained, and possibly the cell count.

Missing observations, such as those in the cellCount column of the Milk table, are said to be *null*. We may require, and the database system may enforce, that all values must be non-*null*, except possibly in the cellCount column. This requirement would not work in the original flat list in Figure 1, because it would prevent us from creating a farm record before the farm has a cow, which is illogical. Furthermore, the splitting of the flat list into separate Farm, Cow and Milk tables means that there is no redundancy and hence less risk of inconsistency: the address of a farm is stated only once per farm, and the farm to which a cow belongs is stated only once per cow.

### Queries in relational databases

The beneficial splitting of the flat list of farm, cow and milk data into three separate tables introduces a challenge, though: How does one combine the tables to obtain useful information, such as the total milk production in each postcode? In a relational database this is done using *queries*, expressed in the language SQL, or Standard Query Language. All modern database systems, including the open source systems MySql and PostgreSql and the commercial systems DB2, Oracle, Microsoft SQL Server and Microsoft Access, understand some variant of SQL and can execute queries involving millions of records in a few seconds. Although the complete SQL language is rather complex, an introduction can be found in any database book, such as [2]. Here we shall just consider some examples of SQL queries, from very simple to moderately complex.

**Figure 5:**
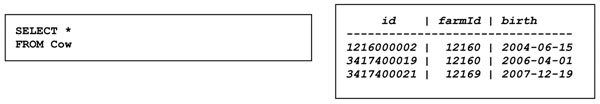
Query to get all columns and rows of the Cow table

The simplest possible query is: To list all cows. Figure 5 shows an SQL query that extracts all columns (denoted by the asterisk *) and all rows of the Cow table; the result, shown in italics to the right, is a "table" very similar to the Cow table itself.

**Figure 6:**
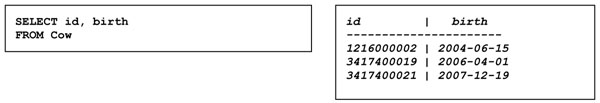
Query to get some columns and all rows of the Cow table

To see only the cow's id and its birth date, we may specify the id and birth columns after SELECT as shown in Figure 6; the result is a table that has only two of the Cow table's columns, but all its rows.

**Figure 7:**
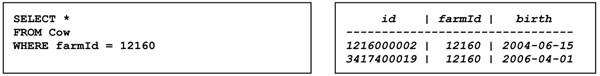
Query to get all columns and some rows of the Cow table

To see only the cows belonging to farm number 12160, we use a WHERE-clause as in Figure 7; the result is a table that has all of the Cow table's columns, but only those of its rows where the cow's farmId equals 12160.

**Figure 8:**
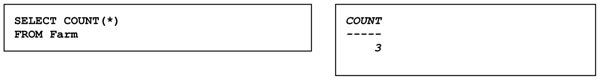
Query to count number of rows in the Farm table

To see just *the number* of farms (rather than the list of all farms) we use *aggregation* by the COUNT function as in Figure 8; the result is still a "table" albeit with a single column and a single row.

**Figure 9:**
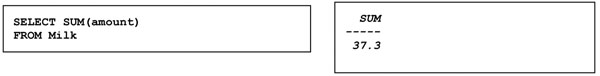
Query to compute total amount of milk over all farms

Similarly, we may compute the total amount of milk by aggregation with the SUM function as shown in Figure 9.

**Figure 10:**
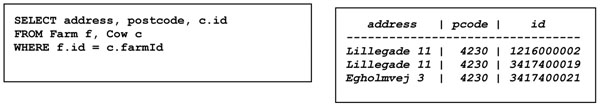
Query to list farms with associated cows

To list each farm (by address) and its cows, we need both the Farm table and the Cow table, but for each farm we are interested only in the cows belonging to that farm. This is called a *join* of the two tables and is illustrated in Figure 10. The join operation in principle considers each combination of a Farm (call it f) and a Cow (call it c) and then the WHERE-clause says that we want only those combinations where the farm's id (that is, f.id) equals the cow's farmId (that is, c.farmId). This may sound cumbersome but can be done very fast in a database system.

**Figure 11:**
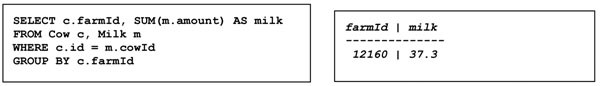
Query to compute total amount of milk for each farm

To compute the total amount of milk for each farm (given by farm id) we again need a join, now between the Cow table and the Milk table. We group the combined records by farm id (using GROUP BY) and use SUM to compute the amount of milk within each group, as shown in Figure 11.

**Figure 12:**
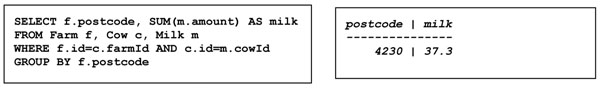
Query to compute total amount of milk for each postcode

To compute the total amount of milk for each postcode we use a three-way join between the Farm, Cow and Milk table, group the records by postcode, and compute the sum within each group by aggregation, as shown in Figure 12.

The above small examples give a taste of some common SELECT queries. Hopefully it transpires that SQL is a very powerful language once one understands how to combine the operations into larger queries. Moreover, relational databases and SQL can be used from inside standard desktop tools such as Excel spreadsheets or the statistical packages R and SAS. Thus large data sets may be stored in a relational database and may be extracted and preprocessed using SQL, and then visualization, statistical analysis and data mining or pattern discovery may be performed using tools that researchers are already familiar with.

### Database design and documentation

The result of a database design is a *database schema*: a list of the database's tables; and for each table, a list of its columns, the type (e.g. number or text) of values in each column, information about which column holds the table's key, which columns are allowed to hold *null* values, and so on.

The database schema is part of the *metadata*, that is, data about the data. Other kinds of metadata that are often neglected, but that are very important for scientific use, are the units of measurements (e.g. liter, kilogram, gram, percentage by volume, percentage by weight), the precision of measurements, time zone information (local time, universal time, daylight savings time), and the exact interpretation of "codes" such as clinical observations (see the section on terminology and ontology) or answer categories of questionnaires. All of this must be documented and the documentation preserved and kept up-to-date for the data to be of any future value.

A central concept in database design is *normal form*, which basically stipulates that tables do not have certain kinds of redundancies. We shall not go into further details here, except to note that the Farm, Cow and Milk tables shown in Figure 2 through Figure 4 are on the so-called Boyce-Codd normal form. Normalization is amply covered in any database book, such as [2].

### Temporal and spatial databases

Our farm-cow-milk database example is highly simplified. In particular, it assumes that a cow belongs forever to the same farm, whereas in reality it may be sold from one farm to another. To solve this problem the Cow table could be made *temporal*, by adding a validFrom and a validTo column. Then each record describes the period in which a given cow belongs to a given farm, which allows for much more detailed queries, such as *what is the number of cows for each farm on 30 July 2010*, or *what is the total milk production per postcode in each of the months of 2010*. Unfortunately, the SQL queries become a good deal more complex. The theory of temporal databases is well-developed; a good introduction is provided by [3].

Moreover, much data is *spatial*: a farm or field is located at a particular place, which may be described by UTM coordinates or longitude and latitude. Knowing *where* objects are *when* allows for queries such as *at what times was this cow near Gelsted* or *find all pairs of cows that were within 8 km of each other at some time* as well as epidemiological analyses and easy visualization.

## Terminology and ontology

**Figure 13:**
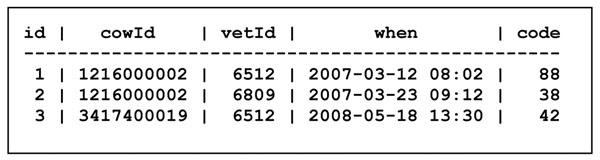
The Clinical table

Here we shall consider a problem that is often overlooked in database books: the design of categories or "codes". Assume that we want to extend our farm-cow-milk database with veterinarians' observations of various diseases of cows. For this purpose we might introduce two more tables. Table Clinical in Figure 13 contains clinical observations about a given cow, made by a given veterinarian at a given time, recording a clinical observation such as *joint infection* by a *code*, here 38.

**Figure 14:**
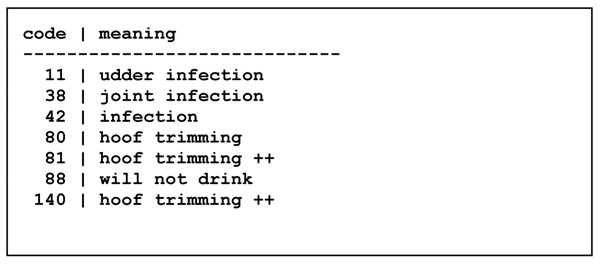
The ClinicalTerm table

Another table, called ClinicalTerm and shown in Figure 14, associates a description with each clinical code.

However, there are some potential problems with the clinical term codes in Figure 14. First of all, codes 81 and 140 appear to have the same meaning, so there is a risk that two people may use different codes for the same observation, which may later produce misleading results (e.g. statistics) when queries are made to the database. Second, no distinction is made between findings (e.g. *88 will not drink*), diagnoses (e.g. *11 udder infection*) and procedures (e.g. *80 hoof trimming*); whether or not this leads to problems depends on the discipline and consistency with which veterinarians register clinical observations. Finally, some codes correspond to subcategories or specializations of others; for instance *11 udder infection* and *38 joint infection* are both special cases of *42 infection*; should one then always use the most specific code available (e.g. 11 or 38) or alternatively always register a more general code (e.g. 42) along with more specific ones (e.g. 11 or 38)? In the former case, will somebody who queries the Clinical table in Figure 13 for all cases of infection remember to also query for the more specific ones (e.g. 11 and 38)? This example illustrates some problems with designing category codes for use in databases, and in classifying observations in general.

A suitable system of "codes", including a consideration about how "codes" relate to each other, is often called a *terminology*, a *controlled vocabulary*, or an *ontology*.

An ontology reflects the domain that it describes, such as the domain of animal disease symptoms discussed above. One must first decide what parts of reality to model (for instance, this cow has an infection), what parts of reality to ignore (such as, where is the infection located). Similarly, in a database of clinical observations one must make clear whether one records symptoms (e.g. diarrhea) or diagnosis (e.g. enteritis) or cause (Salmonella) or all of these. One must also decide how to relate the various parts of reality to each other. For instance, pneumonia is a special case of infection. Moreover, it affects the lungs, which is part of the anatomy. A good domain model should be able to express both forms of hierarchical relationship.

It takes domain experts, technological understanding, and good taste to arrive at adequate domain models that are not too complex.

An example of a well-designed (but complex) domain model is SNOMED/CT, which stands for Systematized Nomenclature of Medical-Clinical Terms. This is a set of standard terms for use in hospitals, electronic patient records, and so on [4]. There are three components of SNOMED/CT:

• Concepts, used to describe disorders (e.g. *128139000 Inflammatory disorder* and *233604007 Pneumonia*), procedures (e.g. *11466000 Cesarean section*), findings (e.g. *62315008 Diarrhea* and *55184003 Infectious enteritis*), causative organisms (e.g. *110378009 Salmonella enterica*), anatomy, and more.

• Descriptions, used primarily for synonyms, e.g. *497137013 Infective enteritis* (synonym for concept *55184003 Infectious enteritis*).

• Relationships, used to describe how concepts relate to each other, e.g. *Pneumonia* IS_A *Inflammatory disorder* and *Pneumonia* FINDING SITE *Lung structure*.

Note how each concept and each description has a unique numeric key. Also note how relationships can be used to relate one concept (pneumonia) both to a disease category and to anatomy, that is, to place the concept in different hierarchies.

SNOMED/CT is maintained by an international organization whose member countries include the United States, United Kingdom, Germany, The Netherlands, Spain, Sweden, Denmark, and many more. In Denmark and most other places, electronic patient records are still based on older and less powerful classification systems, but SNOMED/CT is expected to replace those in the future [5].

Full SNOMED/CT is very complex, with 311,000 concepts, 800,000 descriptions and 1,360,000 relations as of April 2010. A smaller subset for veterinary use is being maintained by Virginia Terminology Services [6].

## Data stewardship, standards, and sharing

Sometimes a whole discipline manages to agree on an ontology, as in the case of SNOMED/CT. Such standardization requires considerable effort, but also offers huge synergistic benefits, especially when databases are made available to all interested parties in a standard format. For instance, within bioinformatics this has led to tremendous advances in research on animals, microorganisms, plants and medicine. Important steps were the 1980es development of standard formats [7] that enable free interchange of DNA sequence data between US, Japanese and European institutions, and the requirement that any sequence data used as basis for a scientific publication must be published, free of any restrictions on further research, in the joint international databases [8].

While the development of standard formats and ontologies is important and enables much better utilization of research investments, it looks more like infrastructure development than research, which means that it appears less exciting and that it may be difficult to obtain funding for it. As a consequence, it may be more tempting to propose new organizations, web sites and portals than to lay the foundation for them, which caused a Nature editorial to admonish that "*Initiatives for digital research infrastructure should focus more on making standardized data openly available*, *and less on developing new portals*" [9].

Thanks to lab automation, sensor development and computerized instruments, research produces new data on a scale never seen before. Yet in many cases the required efforts to document, check and preserve all these data lag behind researchers' ability to generate the data in the first place [10].

This problem is the subject of a report from the US National Academies [11] on *integrity*, *accessibility and stewardship* of digital data, encouraged and sponsored in part by leading journals [12,13]. The report's three main concerns are *integrity* of data (preventing accidental or willful tampering), *sharing* of data (to allow others to check accuracy, verify analyses and build on previous work), and *stewardship* (long-term preservation) of data. Some of the problems have simple technological solutions; for instance, fingerprinting with cryptographic checksums promotes integrity by proving that data has not been tampered with. For the most part however, solutions are organizational and come down to policies and proper *documentation*. Neither sharing nor long-term preservation is very useful if there is confusion about the meaning of code 114, or if some recordings in the same column are in kilograms, others in liters.

To further give a flavour of the report we quote a few of the recommendations:

• Recommendation 1: *Researchers should design and manage their projects so as to ensure the integrity of research data*, *adhering to the professional standards [*...*]*

• Recommendation 6: *In research fields that currently lack standards for sharing research data*, *such standards should be developed [*...*]*

• Recommendation 9: *Researchers should establish data management plans at the beginning of each research project that include appropriate provisions for the stewardship of research data.*

In short, modeling the domain of one's research and designing a database is only the beginning. Researchers must also consider how to preserve and eventually share raw data to enable replication of experiments and statistical analyses as well as future research that may use the data in unanticipated ways.

## Competing interests

The authors declare that they have no competing interests.
